# Mentalization-Based Treatment in Groups for Adults With Autism Spectrum Disorder

**DOI:** 10.3389/fpsyg.2021.708557

**Published:** 2021-08-12

**Authors:** Katharina Krämer, Annekatrin Vetter, Ulrich Schultz-Venrath, Kai Vogeley, Sophia Reul

**Affiliations:** ^1^Department of Psychiatry, University Hospital Cologne, Cologne, Germany; ^2^Rheinische Fachhochschule Köln, University of Applied Sciences, Cologne, Germany; ^3^Private Practice for Psychosomatic Medicine and Psychotherapy, Cologne, Germany; ^4^University of Witten/Herdecke, Witten, Germany

**Keywords:** mentalization-based treatment, autism spectrum disorder, group therapy, mentalizing, theory of mind, Asperger's syndrome

## Abstract

In order to successfully interact with others in social encounters, we have to be attentive to their mental states. This means, we have to implicitly and explicitly interpret our own actions as well as the actions of others as meaningful on the basis of the ascription of intentional mental states. However, this ability, often referred to as mentalizing, seems to be impaired in autism spectrum disorder (ASD). Individuals with ADS show specific deficits relating to the representation of mental states of others. Especially, the spontaneous, intuitive attribution of and reaction to others' mental states seem to be impaired. Mentalization-Based Treatment (MBT) is a form of psychotherapy in individual and group settings that focuses on the education and enhancement of mentalizing. Although the scope of MBT is broad and MBT has been already proven to be useful in a variety of mental disorders, no attempt has been made to apply MBT in patients with ASD. In our study, we adapted MBT for adults with ASD in a therapeutic group setting to examine the feasibility as well as the effectiveness of the treatment in this patient group. During 15–20 weeks of weekly group therapy, we surveyed the patients' acceptability of the intervention. Additionally, changes in mentalizing difficulties were measured before and after treatment. Results show a high acceptance of the treatment and an improvement in the patients' mentalizing abilities, presenting MBT as a promising treatment option for ASD.

## Introduction

The ability to implicitly and explicitly interpret our own actions as well as the actions of others as meaningful on the basis of the ascription of intentional mental states is crucial for successful social encounters. In Autism spectrum disorder (ASD), however, this ability, often referred to as mentalizing or Theory of Mind (ToM), seems to be impaired (Baron-Cohen, 1997, 2000; Frith, 2003). ASD is a neurodevelopmental disorder characterized by deficits in social communication and social interaction as well as repetitive patterns of behavior, interests or activities (American Psychiatric Association, 2013). Qualitative impairments in social communication and reciprocal interaction, which often lead to misunderstanding others (White et al., 2009; Brewer et al., 2017; Tanu and Kakkar, 2019), are closely linked to specific mentalizing deficits in ASD. Although ASD patients without cognitive impairment often develop strategies to compensate for their deficits and to manage social interactions (David et al., 2010), even adults with ASD without intellectual impairment still show specific deficits in spontaneous, intuitive attribution of and reaction to others' mental states in complex everyday situations (Frith, 2003; Kuzmanovic et al., 2011). These mentalizing deficits might further contribute to the disproportionate risk for developing mental health comorbidities in ASD, such as anxiety and depression (Hollocks et al., 2019). Therefore, psychotherapeutic treatment is crucial for this patient group. As ASD is not causally treatable, psychotherapeutic interventions essentially focus on the improvement of life quality and the extension of patients' behavioral repertoire (Remschmidt and Kamp-Becker, 2006; Gawronski et al., 2011). Interestingly, treatments that particularly focus on the improvement of mentalizing abilities, such as Mentalization-Based Treatment (MBT) (Karterud, 2015; Bateman and Fonagy, 2016), are not usually part of ASD therapy programs. Instead, psychotherapy research of ASD has mainly focused on cognitive behavioral therapy (CBT) in individual and group settings (Walters et al., 2016; Dziobek and Stoll, 2019; Spain and Happé, 2020; Mayer-Benarous et al., 2021). To the best of our knowledge, MBT has never been applied in this particular patient group until today.

MBT is a manualized psychotherapeutic treatment which specifically focuses on improving patients' capacity to mentalize (Karterud, 2015; Bateman and Fonagy, 2016). It was originally developed for the treatment of borderline personality disorder (Bateman and Fonagy, 2004, 2008), but has since then been applied to a variety of mental disorders and clinical presentations (Malda-Castillo et al., 2019) such as depression (Jakobsen et al., 2014), eating disorders (Robinson et al., 2016), self-harm (Rossouw and Fonagy, 2012), and psychotic disorders (Weijers et al., 2016) in therapeutic individual and group settings (Schultz-Venrath and Felsberger, 2016). According to Bateman and Fonagy (2016), well-functioning mentalizing can be described as a balance along four dimensions: Automatic vs. explicit mentalizing, mentalizing the self vs. others, cognitive vs. affective mentalizing, and mentalizing with regard to internal vs. external features. Imbalances along these dimensions can lead to psychological distress and can furthermore be associated with different types of psychopathology (“mentalizing profile”) (Bateman and Fonagy, 2016). In a previous article we proposed a mentalizing profile for ASD (Reul et al., 2020), suggesting that ASD individuals often show explicit mentalizing in social encounters, as they have difficulties concerning the spontaneous, intuitive ascription of mental states. Also, they focus on the self as well as on cognitive aspects of mentalizing. Corresponding to their impairment in the perception of non-verbal cues, they are less sensitive to external cues such as subtle facial expressions, eye movements or tone of voice.

We adapted MBT for adults with ASD in a therapeutic group setting (MBT-ASD) in order to examine the feasibility as well as the effectiveness of the treatment in this patient group. Therefore, we collected both qualitative and quantitative data from two different therapy groups running over 15–20 weeks to analyze and evaluate the therapeutic process as well as the outcome. Here, we focus on the quantitative data to answer two main questions: Do patients with ASD accept MBT and do they benefit from the treatment?

## Materials and Methods

### Ethics Statement

The study was conducted with the approval of the local ethics committee of the Medical Faculty of the University of Cologne, Germany. Participants gave their written informed consent before taking part.

### Participants

A total of 18 patients with Asperger's syndrome (AS), a form of ASD associated with no general delay in language or cognitive development, participated in the study. Participants were recruited via email to all patients diagnosed in the autism outpatient clinic at the Department of Psychiatry at the University Hospital Cologne, Germany, who indicated their overall willingness to participate in research studies. As part of a systematic assessment of the autism outpatient clinic, AS diagnoses were made independently by two specialized physicians corresponding to ICD-10 criteria and were supplemented by an extensive neuropsychological assessment. MBT treatment was delivered in two patient groups. Allocation to the two MBT treatment groups (MBT-G1 and MBT-G2) depended on the time participants responded to the email. Participants of MBT-G1 and MBT-G2 did not differ with respect to age, Autism Spectrum Quotient (AQ) (Baron-Cohen et al., 2001), Empathy Quotient (EQ), Systemizing Quotient-Revised (SQ) (Wheelwright et al., 2006) and intelligence (see Table 1). Intelligence was assessed by a German multiple choice vocabulary test (“Wortschatztest,” WST) (Schmidt and Metzler, 1992), which allows a quick and valid estimation of general intelligence (Lehrl et al., 1995; Suslow, 2009).

**Table 1:** Demographic and clinical variables.

	**MBT-G1** **(***n*****=** 8)**		**MBT-G2** **(***n*****=** 8)**		**Statistics**	
	***M***	**SD**	***M***	**SD**		
Gender (m:f)	4:4		6:2			
Age (y)	43.50	8.54	46.63	12.58	*t*_(14)_ = −0.58	*p* = 0.570
AQ	41.14	4.60	43.50	3.21	*t*_(13)_ = −1.16	*p* = 0.265
EQ	12.43	5.97	12.00	5.24	*t*_(13)_ = 0.15	*p* = 0.884
SQ	47.14	14.25	46.88	9.94	*t*_(13)_ = 0.04	*p* = 0.967
IQ	110.63	10.86	115.00	5.88	*t*_(14)_ = −1.00	*p* = 0.333

*M, mean; SD, standard deviation; m, male; f, female; y, years; AQ, Autism Spectrum Quotient (Cut-off > 26); EQ, Empathy Quotient (Cut-off < 30); SQ, Systemizing Quotient-Revised*.

*AQ, EQ, and SQ scores of one participant are missing*.

Both groups initially consisted of nine participants. In MBT-G1 one participant dropped out after 6 weeks of treatment due to simultaneous occupational commitments. In MBT-G2 one participant dropped out after the first session due to the extensive traveling time. After dropout both groups consisted of eight participants (MBT-G1: 4 male, mean age 43.50, SD = 8.54 years; MBT-G2: 6 male, mean age 46.63, SD 12.58 years). In MBT-G1 four participants reported a comorbidity of depression, three of those were taking antidepressant medication. Six participants in MBT-G2 reported a comorbidity of depression, four of those were taking antidepressant medication. In MBT-G1 one of the depressive patients additionally suffered from an anxiety disorder. ADHD was reported for one patient in every group. Experiences with individual psychotherapeutic treatment in the past was reported for all participants in MBT-G1 (CBT *n* = 5; Psychodynamic treatment *n* = 2; not specified *n* = 1). In MBT-G2 seven participants underwent individual psychotherapy in the past (CBT *n* = 6; Psychodynamic treatment *n* = 1). Five participants in MBT-G1 and seven participants in MBT-G2 received previous therapeutic treatment at the autism outpatient clinic at the Department of Psychiatry at the University Hospital Cologne, Germany (CBT group therapy for ASD) (Gawronski et al., 2011, 2013).

### Procedure

For MBT-ASD, we adapted existing MBT and MBT-G programs (Karterud, 2015; Bateman and Fonagy, 2016) for adults with ASD in a therapeutic group setting. Before the treatment, an initial anamnestic interview was conducted with one of the therapists (KK, SR). During this interview, participants formulated therapy goals and had the opportunity to ask questions about the procedure. During four introductory MBT-ASD sessions participants were educated about ASD, mentalizing, emotions, and MBT methods. In order to improve and stimulate patients' ability to consciously represent their own and others' mental states and feelings in everyday situations, MBT-ASD focused on engaging group members in mentalizing internal and external events in the therapy sessions. Therapists curiously explored patients' narratives (“not-knowing stance”) while regulating group phases (opening phase, middle working phase, termination phase) and managing group boundaries (e.g., starting and ending sessions punctually) (Karterud, 2015; Reul et al., 2020). MBT-ASD originally consisted of 20 weeks of weekly group therapy (MBT-G1, October 2019—March 2020). However, due to the constraints of the Covid-19 pandemic, MBT-G2 had to be shortened to 15 weeks (August 2020—November 2020). All sessions lasted for 90 min and were audio- and videotaped. Two psychodynamic therapists (KK, SR) that received a 3-day MBT training (MBT: Basic Training, Heidelberg, Germany) conducted the treatment. Additionally, monthly group supervision was provided by an experienced MBT-G therapist (USV). In order to ensure adherence, each session was also assessed (AV) using the MBT Adherence and Competence Scale (MBT-ACS) (Karterud et al., 2013). Data were collected at baseline (T0), after the introductory sessions (T1), and at the end of the treatment (T2).

### Measures

Patients' acceptability of the treatment was assessed with the German version of the *Helping Alliance Questionnaire* (HAQ) (Bassler et al., 1995). Aside from a global measure of patients' perception of the quality of the working alliance with the therapist (12 items; HAQ), two subscales, “relation to the therapist” (6 items; HAQ-1) and “satisfaction with the therapeutic outcome” (5 items; HAQ-2), were computed. Also, item 12 yielded information on patients' perceived overall success of the therapy (HAQ-I-12). The *HAQ* was used after the introductory sessions (T1), in order to ensure the patients' ability to assess the therapeutic relationship (Bassler et al., 1995), and at T2. Finally, at the end of the treatment, participants indicated whether they were interested in further participation of MBT-ASD, if it would be continued as a treatment option at the autism outpatient clinic.

To evaluate the effectiveness of MBT-ASD we explored mentalizing abilities as well as the extent of psychological distress at baseline and at the end of the treatment. In the present study, mentalizing was assessed with a video-based test of subtle mindreading difficulties, the *Movie for the Assessment of Social Cognition* (MASC) (Dziobek et al., 2006). It was developed to evaluate the specific mentalizing difficulties of patients with ASD and involves watching a short film (15 min) and answering questions referring to the actors' mental states. A self-report measure for alexithymia, the *Toronto Alexithymia Scale* (TAS-20) (Bagby et al., 1994), was used to assess patients' deficiencies in identifying and describing emotions experienced by one's self and others at baseline and at the end of the treatment. Also, as depression is a common comorbidity in ASD (Stewart et al., 2006; Hollocks et al., 2019) and several AS patients of the current study also suffered from depression, we assessed changes in the severity of depression with the German edition of the *Beck Depression Inventory* (BDI) (Hautzinger et al., 1995). Furthermore, a German short version of the Symptom-Checklist-90-R(SCL-90-R), the “*Symtom-Checkliste-Kurzversion-9*” (SCL-K-9) (Klaghofer and Brähler, 2001) consisting of nine items that cover all original subscales of the SCL-90-R, was used to measure the progress and outcome of the treatment on a global level of symptomatology (Müller et al., 2010).

### Data Analyses

All data were analyzed using IBM SPSS Statistics 26 (SPSS Inc, Chicago, IL, 2019). As assumptions for parametric tests were fulfilled (Gaussian distribution, homoscedasticity), independent *t*-tests were used to analyze differences between the patient groups MBT-G1 and MBT-G2 in MASC, TAS-20, BDI, and SCL-K-9 scores at baseline and at the end of the treatment. Furthermore, independent *t*-tests were used to analyze differences between MBT-G1 and MBT-G2 for HAQ scores at T1 and T2. Subsequently, dependent *t*-tests were conducted to analyze changes in participants' scores before and after the treatment. For MASC, TAS-20, BDI and SCL-K-9 we compared scores between T0 and T2. Changes in HAQ, HAQ-1, HAQ-2, and HAQ-I-12 scores between T1 and T2 were also computed with dependent *t*-tests. Influences of gender and age were analyzed with covariance analyses. Cohen's *d* is reported as a measure of effect size. The following conventions for interpreting *d* are suggested: Small effect: *d* = 0.2; medium effect: *d* = 0.5; large effect: *d* = 0.8 (Cohen, 1988).

## Results

We compared both groups to test whether we could combine them for further analysis. Participants of MBT-G1 and MBT-G2 did not differ in the scores of MASC at T0 [*t*_(14)_ = −2.14, *p* = 0.052] and T2 [*t*_(14)_ = −1.30, *p* = 0.215], TAS-20 at T0 [*t*_(14)_ = 1.52, *p* = 0.150] and T2 [*t*_(14)_ = 1.46, *p* = 0.167], BDI at T0 [*t*_(14)_ = 0.15, *p* = 0.883] and T2 [*t*_(14)_ =0.83, *p* = 0.422], and SCL-K-9 at T0 [*t*_(14)_ = 1.58, *p* = 0.138] and T2 [*t*_(14)_ = 0.90, *p* = 0.382]. Furthermore, no differences were found in the scores of HAQ at T1 [*t*_(14)_ = 1.87, *p* = 0.082] and T2 [*t*_(14)_ = 0.48, *p* = 0.642] between MBT-G1 and MBT-G2. Hence, results are reported for the whole group (*N* = 16) (see Table 2).

**Table 2:** Measures of acceptance and effectiveness of MBT-ASD.

	**T0 *M* (SD)**	**T1 *M* (SD)**	**T2 *M* (SD)**	**df**	***t***	***p***	***d***
HAQ		47.06 (5.91)	50.88 (5.12)	15	−1.95	0.070	
HAQ-1		28.47 (2.96)	29.50 (4.18)	15	−0.74	0.466	
HAQ-2		18.59 (3.59)	21.38 (2.85)	15	**–** ***2.68***	0.017^*^	***0.67***
HAQ-I-12^a^		3.52 (1.03)	2.31 (0.87)	15	***4.14***	0.001^**^	***1.03***
MASC	28.25 (7.02)		32.56 (5.70)	15	**–** ***3.31***	0.005^**^	***0.83***
TAS-20	61.00 (9.93)		57.25 (9.61)	15	***2.65***	0.018^*^	***0.66***
BDI	14.56 (8.07)		18.06 (9.28)	15	**–** ***2.25***	0.040^*^	***0.56***
SCL-K-9	11.00 (5.99)		12.88 (7.15)	15	−1.42	0.176	

*M, mean; SD, standard deviation; HAQ, Helping Alliance Questionnaire; HAQ-1, Helping Alliance Questionnaire: subscale 1 (relation to the therapist); HAQ-2, Helping Alliance Questionnaire: subscale 2 (satisfaction with the therapeutic outcome); HAQ-I-12, Helping Alliance Questionnaire: Item 12 (therapeutic success); MASC, Movie for the Assessment of Social Cognition; TAS-20, Toronto Alexithymia Scale (Cut-off > 54); BDI, Beck Depression Inventory (scores from 10 to 18 indicate mild to moderate depression); SCL-K-9, Symtom-Checkliste-Kurzversion-9*.

*Effect size (d) is only reported for significant results*.

a *Lower scores indicate higher perception of therapeutic success*.

* *p < 0.05*,

** *p < 0.01*.

*Bold values indicate significant changes from T0/T1 to T2*.

Results show that HAQ scores did not significantly change between T1 (*M* = 47.06, SD = 5.91) and T2 (*M* = 50.88, SD = 5.12), indicating that participants' overall acceptability of the treatment was stable throughout the therapy process [*t*_(15)_ = −1.95, *p* = 0.070]. Also, participants' positive perception of the relation to the therapists (HAQ-1) did not significantly differ at T1 (*M* = 28.47, SD = 2.96) and T2 [*M* = 29.50, SD = 4.18, *t*_(15)_ = −0.74, *p* = 0.466]. Satisfaction with the therapeutic outcome (HAQ-2) significantly increased between the introductory sessions (*M* = 18.59, SD = 3.59) and at the end of ASD-MBT [*M* = 21.38, SD = 2.85, *t*_(15)_ = −2.68, *p* = 0.017, *d* = 0.67]. Furthermore, participants' perception of the therapeutic success (HAQ-I-12) increased between T1 (*M* = 3.52, SD = 1.03) and T2 [*M* = 2.31, SD = 0.87, *t*_(15)_ = 4.14, *p* = 0.001, *d* = 1.03]. Finally, almost all participants (*n* = 15) indicated that they were interested in further participation of MBT-ASD, if it would be continued as a treatment option at the autism outpatient clinic. Only one participant (MBT-G2) negated the question.

**Figure 1:**
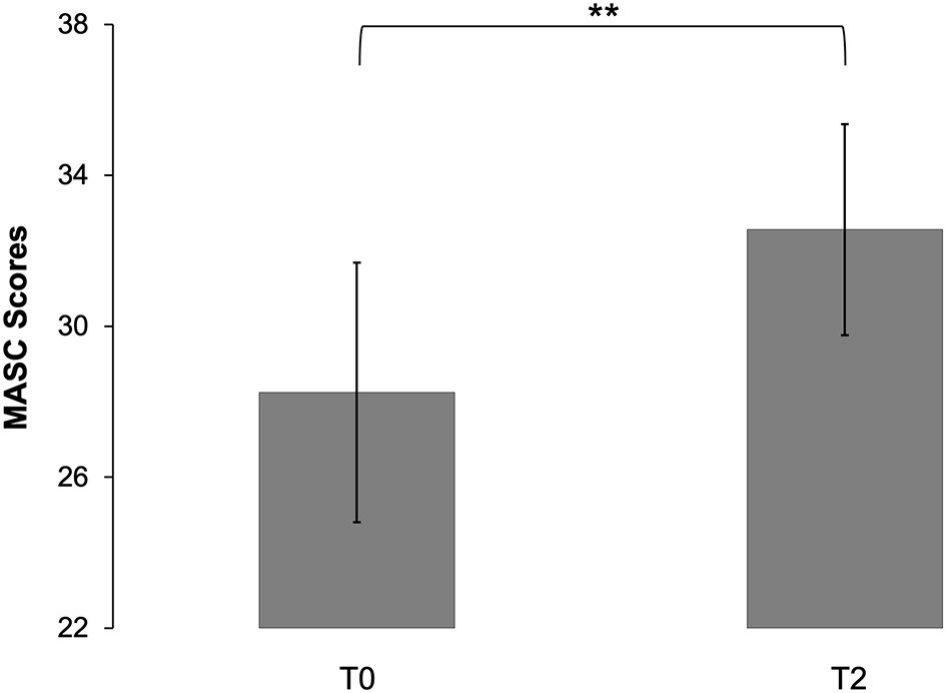
Changes in mentalizing abilities from T0 to T2 measured with the Movie for the Assessment of Social Cognition (MASC). Error bars indicate the 95% confidence interval; ^**^p < 0.01.

AS patients showed an enhancement in mentalizing abilities assessed with the MASC at the end of the treatment (*M* = 32.56, SD = 5.70) compared to the beginning [*M* = 28.25, SD = 7.02, *t*_(15)_ = −3.31, *p* = 0.005, *d* = 0.83] (see Figure 1). TAS-20 scores significantly differed at baseline (*M* = 61.00, SD = 9.93) compared to the end of the treatment (*M* = 57.25, SD = 9.61), indicating that patients' ability to identify and describe emotions experienced by one's self and others increased during the course of MBT-ASD [*t*_(15)_ = 2.65, *p* = 0.018, *d* = 0.66]. Covariance analyses showed no influence of gender or age on MASC scores [gender: *F*_(1)_ = 1.924, *p* = 0.189; age: *F*_(1)_ = 1.409, *p* = 0.257] and TAS-20 scores [gender: *F*_(1)_ = 2.136, *p* = 0.168; age: *F*_(1)_ = 4.516, *p* = 0.053]. However, patients showed enhanced BDI scores at T2 (*M* = 18.06, SD = 9.28) compared to T0 (*M* = 14.56, SD = 8.07), which suggests an increase of the depressive symptomatic [*t*_(15)_ = −2.25, *p* = 0.040, *d* = 0.56]. SCL-K-9 scores did not differ at T2 (*M* = 12.88, SD = 7.15) and T0 [*M* = 11.00, SD = 5.99, *t*_(15)_ = −1.42, *p* = 0.176].

## Discussion

The present study sought to investigate the feasibility as well as the effectiveness of MBT in a group setting for adults with ASD. In this brief report we focus on two main questions: To determine if patients with ASD accept the MBT setting and if they benefit from this psychotherapeutic approach.

The first question is linked to the observation that the therapeutic setting of MBT—coming from a psychodynamic background—is less structured than CBT settings, which have already been proven to be successful in the treatment of ASD (Walters et al., 2016; Spain and Happé, 2020). In contrast to CBT settings, in MBT sessions therapists work without scheduled topics, worksheets, role plays or a fixed speaking order. As the sessions are neither structured by a manual nor the therapists, participants themselves have to spontaneously agree upon the content of the session and their individual involvement (e.g., speaking time, sharing of experiences) in the format of an open discussion. Getting used to a specific group dynamic and finding an individual role in the group process is challenging for every therapy participant, however, this is considered a necessary component of a successful therapeutic process (Yalom and Leszcz, 2020). But taking into account that patients with ASD are especially focused on structure and rules, particularly in social situations, this might cause an unusually high amount of stress and insecurity for this specific group of patients. Thus, our first concern was whether ASD participants felt comfortable to engage with the therapists and each other in such a comparably unstructured therapeutic setting.

Interestingly, our results show a high acceptance of the MBT setting. Participants evaluated the therapeutic relationship on an unaltered, constantly high level (HAQ-1) while their satisfaction with the treatment (HAQ-2) and their evaluation of the therapeutic success (HAQ-I-12) grew from the first therapeutic session (T1) to the last (T2). In order to be responsive to ASD participants' need for structure and rules and to build a fundament of trust and transparency, we developed a detailed and structured entering process for MBT-ASD. During an initial individual interview, participants had the opportunity to get basic information and ask questions about the treatment procedure. Also, we developed four structured introductory sessions before the actual treatment which included psychoeducation on ASD and emotions, information on MBT, and a detailed discussion about patients' behavior during sessions (e.g., confidentiality agreement, how to address each other, seating plan). On this basis, we created an atmosphere where ASD participants felt comfortable during the less structured therapeutic MBT sessions. These considerations are supported by the observation that participants already showed a high satisfaction with the therapeutic relationship at the first HAQ-1 rating after the introductory sessions, which remained stable throughout the whole therapy process. Taken together, HAQ results indicate a high level of acceptance for MBT among participants. This is supported by the fact that almost all participants stated that they were interested in further participation of MBT-ASD, if it would be continued as a treatment option at the autism outpatient clinic.

Our second aim for this report was to investigate, whether patients with ASD benefit from MBT. To answer this question we evaluated patients' mentalizing ability with an instrument specialized to detect ToM deficits in ASD (Dziobek et al., 2006). Although the MASC was not specifically developed to evaluate training effects of social cognition, we decided to use it to estimate the effect of the treatment on mentalizing abilities, as it has recently shown to be a promising tool to measure social cognition enhancement in MBT research (Steinmair et al., 2021). Interestingly, our results show a significant improvement over the course of the treatment. Regarding the fact that ASD is not causally treatable, enhancing mentalizing abilities through MBT might still be a promising method to help AS patients to better understand and reflect interpersonal situations. We presume that specific MBT interventions (Karterud, 2015; Reul et al., 2020), like the constant invitation of mentalizing internal and external events by the therapist, helps participants to adapt to social situations. Also, they are enabled to use the therapist as a role model to tackle social situations from a more curious point of view, by adapting the “not-knowing stance.” This might lead to more self-confidence and security in social interactions and could also explain the changes we found in the self-report measure for alexithymia. Although ASD patients still showed pathological ratings (TAS-20 M = 57, Cut-off = 54) they evaluated their own ability to identify and describe emotions experienced by oneself and others significantly better after than before the treatment. After getting first evidence on the therapeutic outcome of MBT-ASD, future research should specifically focus on the therapeutic MBT process in a more fine-grained manner to better understand why its interventions are helpful for ASD individuals and how MBT affects ASD patients' experiences and behavior. Previous MBT studies for different mental disorders have mostly focused on evaluating the improvement of clinical factors and specific core symptoms (e.g., emotional control for BPD). Therefore, we cannot directly compare our results with other findings. However, one study has identified patients' social cognition capacities (measured with the MASC) as an important predictor for successful MBT treatment (Kvarstein et al., 2020). This indicates that social cognition and ToM are important factors for successful therapeutic work and mental health in general. Accordingly, it seems even more important to specifically enhance ASD patients' mentalizing abilities, in order to facilitate effective treatment of associated clinical symptoms (e.g., depression, anxiety).

Even if the global level of symptomatology (SCL-9-K) did not change over the treatment, we unfortunately found an increase in depressive symptoms (BDI). To understand this effect, some further aspects have to be examined. First, depression scores only increased for patients that already suffered from clinically relevant depression before the treatment (MBT-G1 *n* = 4; MBT-G2 *n* = 6). Furthermore, mood deterioration can be explained as a side-effect of psychotherapy: increase of symptoms often occur during a psychotherapeutic process caused by different reasons (Berk and Parker, 2009). Especially at the beginning of the treatment, psychological defense mechanisms get weakened (e.g., through higher introspection, focus on feelings and conflicts) and patients get more aware of their distress and harm (Linden, 2013). Taking into account that 15 or 20 sessions is a short-time therapy cycle and MBT is not explicitly developed for resource activation only, it might be helpful to extend the cycle to a higher number of sessions to absorb possible side-effects over time. Patients in previous studies that investigated the effectiveness of MBT for combined personality disorder and antisocial personality disorder (Bateman et al., 2016), eating disorders (Robinson et al., 2016) and self-harm in adolescents (Rossouw and Fonagy, 2012), for example, showed higher overall symptom reduction. In these studies, treatment periods were considerably longer (12–18 months).

Another external factor influencing the increase of depressive symptoms might be the ongoing Covid-19 pandemic, which affected both groups. Patients' narratives during the sessions clearly showed that they suffered from this special situation. Home office, remote work (e.g., phone and video conferences), wearing of face-masks, social isolation and a huge amount of new and ever-changing rules prevented them from following their routines and caused high levels of stress. Even though there is no statistical proof for this assumption, we still think that it should be considered in the interpretation of the results. There is increasing evidence that particularly people with recurring mental health issues or psychological illness in the past have come down with clinically relevant symptoms since the Covid-19 pandemic (Yao et al., 2020).

## Limitations

A main limitation of the present study is the small sample size, which makes our results preliminary. Also, as there hasn't been a follow-up evaluation yet to observe the sustainability of the therapeutic effects, a final evaluation of the effectiveness of the treatment remains open. Furthermore, we did not investigate the influence of therapist effects (e.g., gender, age, personality, experience) (Delgadillo et al., 2020) in the present study.

Additionally, the treatment cycle of 15–20 weeks of weekly group therapy is relatively short. Caused by the constraints of the Covid-19 Pandemic the cycle length also differed slightly between the groups (MBT-G1 20 sessions, MBT-G2 15 sessions). However, group results did not significantly differ. For future research we suggest an extended cycle of 40 sessions to ensure a stabilization of therapeutic effects.

Finally, our groups showed a high homogeneity considering age and diagnosis. We only treated adults with AS that showed no cognitive impairment. Therefore, results are not generalizable to all persons with ASD including persons with learning disabilities.

## Conclusion

Overall, results show a high acceptance of the treatment and a significant improvement of patients' mentalizing abilities, indicating that MBT-ASD is a feasible and promising treatment option for ASD. In particular, we want to emphasize the importance of a detailed patient introduction before the actual therapeutic MBT sessions. Although our data is still preliminary, MBT-ASD shows a high potential to sustainably expand the therapeutic landscape for adults with ASD, considering the lack of evidence-based therapeutic treatment for this specific patient group.

## Data Availability Statement

The raw data supporting the conclusions of this article will be made available by the authors, without undue reservation.

## Ethics Statement

The studies involving human participants were reviewed and approved by Ethics committee of the Medical Faculty of the University of Cologne, Germany. The patients/participants provided their written informed consent to participate in this study.

## Author Contributions

KK conducted MBT-ASD and contributed to the patient acquisition and diagnostic process, development of the study concept and design, drafting of the manuscript, analysis and interpretation of the data, and study coordination. AV was involved in the acquisition of the adherence data and revising of the manuscript. US-V conducted supervision and was involved in revising the manuscript. KV contributed to the patient acquisition and diagnostic process and revising of the manuscript. SR conducted MBT-ASD and contributed to the development of the study concept and design, drafting of the manuscript, interpretation of the data, and study coordination. All authors read and approved the final manuscript.

## Conflict of Interest

The authors declare that the research was conducted in the absence of any commercial or financial relationships that could be construed as a potential conflict of interest.

## Publisher's Note

All claims expressed in this article are solely those of the authors and do not necessarily represent those of their affiliated organizations, or those of the publisher, the editors and the reviewers. Any product that may be evaluated in this article, or claim that may be made by its manufacturer, is not guaranteed or endorsed by the publisher.
